# THE EMPOWERED CAREGIVER: A SCALABLE AND PRAGMATIC EVIDENCE-BASED PROGRAM FOR DEMENTIA CAREGIVERS

**DOI:** 10.1093/geroni/igae098.1899

**Published:** 2024-12-31

**Authors:** Katherine Judge, Claire Grant, Lauren Stratton, Monica Moreno, Sam Fazio

**Affiliations:** Cleveland State University, Cleveland, Ohio, United States; Cleveland State University, Cleveland, Ohio, United States; Alzheimer’s Association, Chicago, Illinois, United States; Alzheimer’s Association, Chicago, Illinois, United States; Alzheimer’s Association, Chicago, Illinois, United States

## Abstract

Informal caregivers provide the bulk of care to older adults with dementia. Few studies have evaluated pragmatic and scalable on-line educational programs for caregivers. An RCT (N=247) was used to evaluate a new program, Empowered Caregiver, across 3 timepoints (T1/baseline; T2 after program completion; and T3 45 days after T2.) The program included two 90-minute sessions delivered online via community educators and addressed communication, dementia-related behaviors, caregiver stress/support, and person-centered care. Materials provided pragmatic and easy to implement caregiver strategies. Program acceptability also was assessed. Participants were M=46.41 years old (SD=16.23), female (63.6%), Caucasian (54.3%), married (47%), heterosexual (80.2%), and worked part- or full-time (50.4%). Separate 2 (condition) x 3 (time) repeated measures GLMs were conducted. Program participants (n=131), compared to control (n=116), reported significant improvements with: Caregiver Self-Efficacy, Emotional Health Strain, Role Captivity, Caregiver Mastery, Personal Gain, Unmet Needs, Unmet Needs Distress, Anxiety Symptoms, Depression Symptoms, Behavioral Intentions, and Behavioral Actions. Mean ratings of program acceptability using a 5-point Likert scale ranged from 4.17 to 4.58. Participants indicated they would very likely recommend the program to others (91.5%). Results indicated the Empowered Caregiver program was efficacious in improving a wide range of caregiver outcomes along with being highly acceptable. Overall, findings address a significant gap in the literature and a current limitation in providing caregivers with timely evidence-based information, tools, and resources. Discussion will highlight key implementation issues, alignment of program dosage with measures, the need for pragmatic and scalable programs, and future directions.

